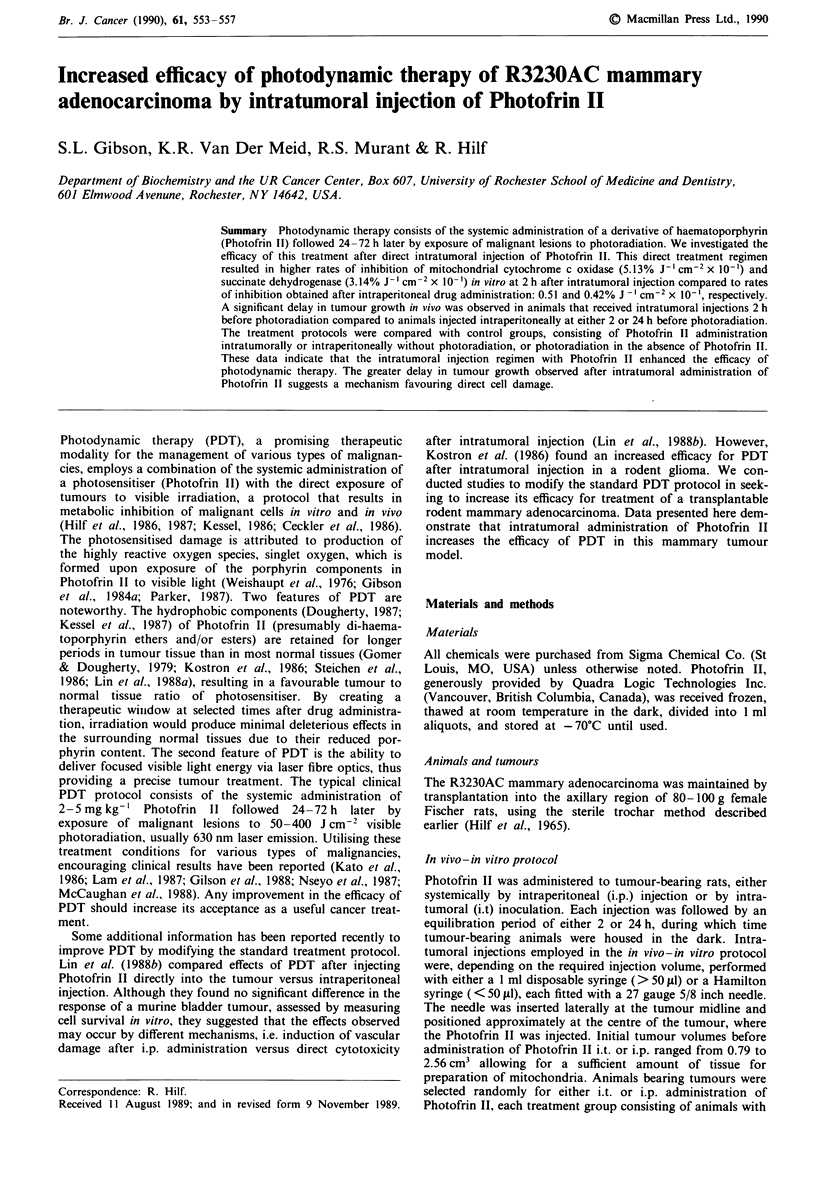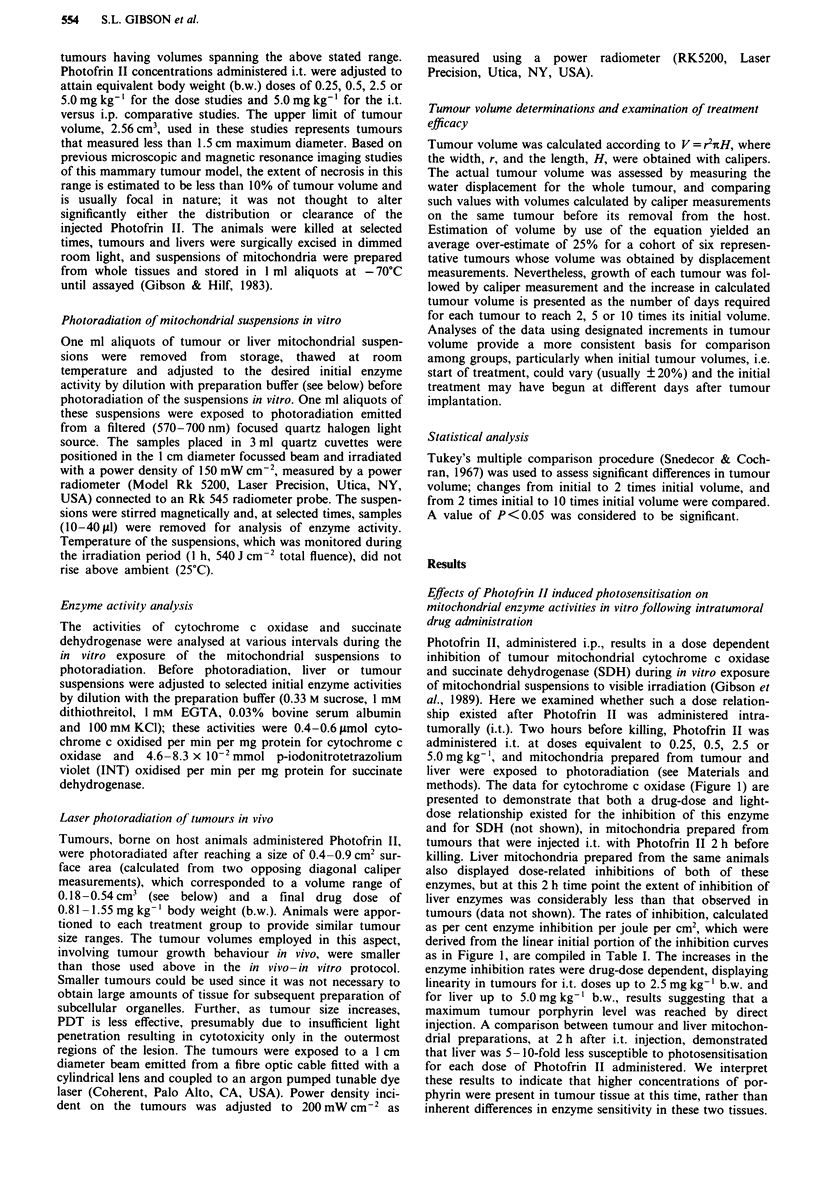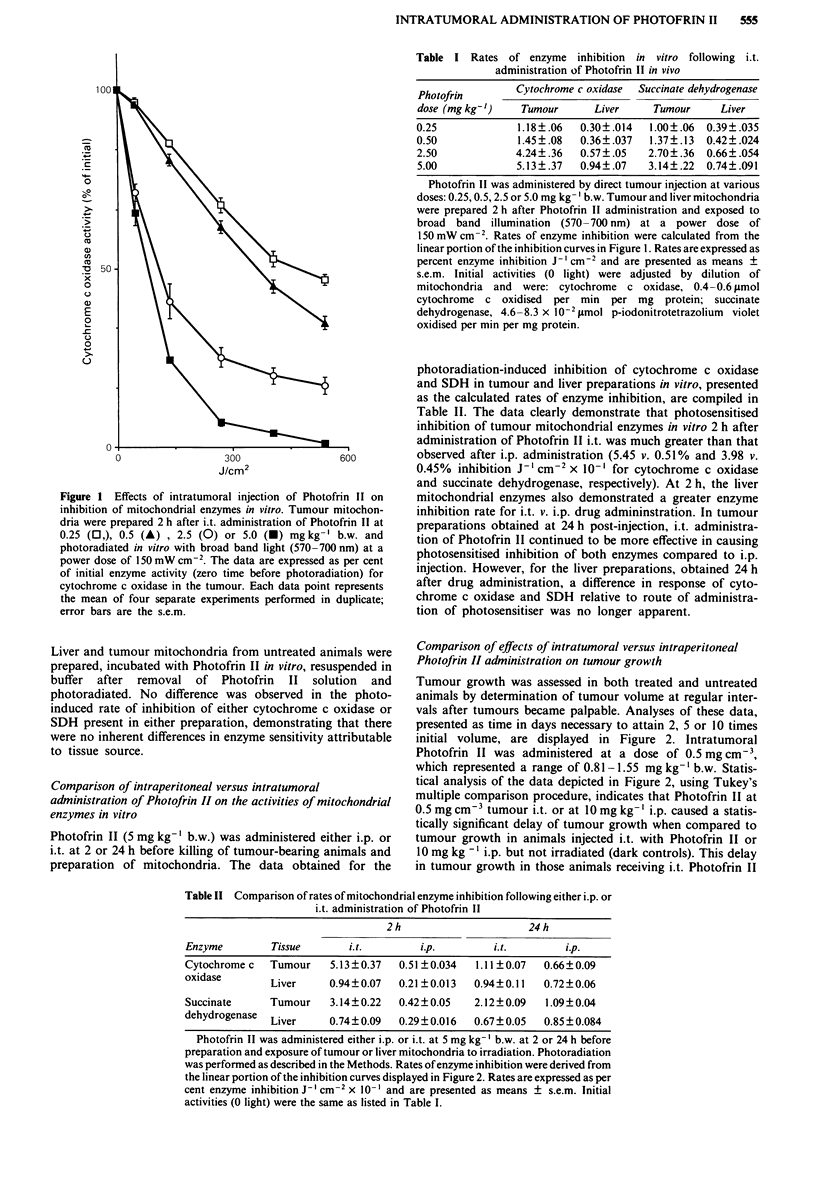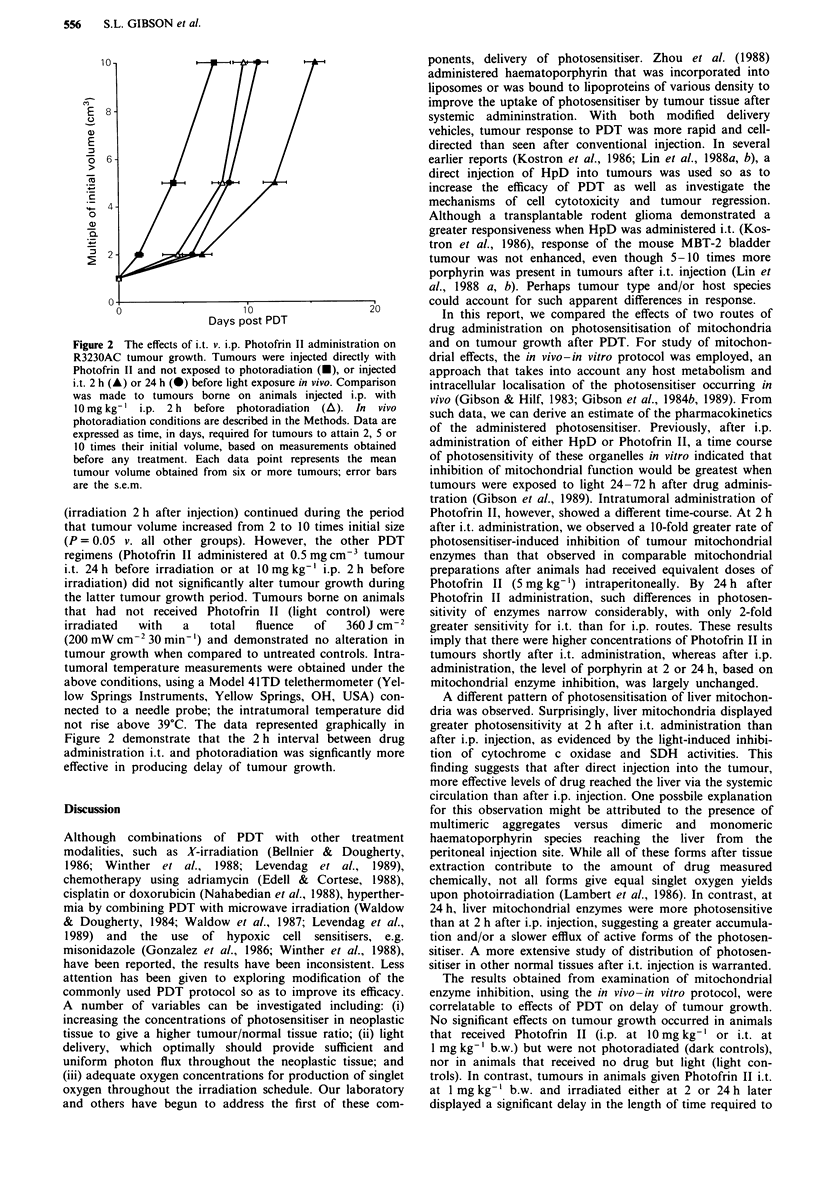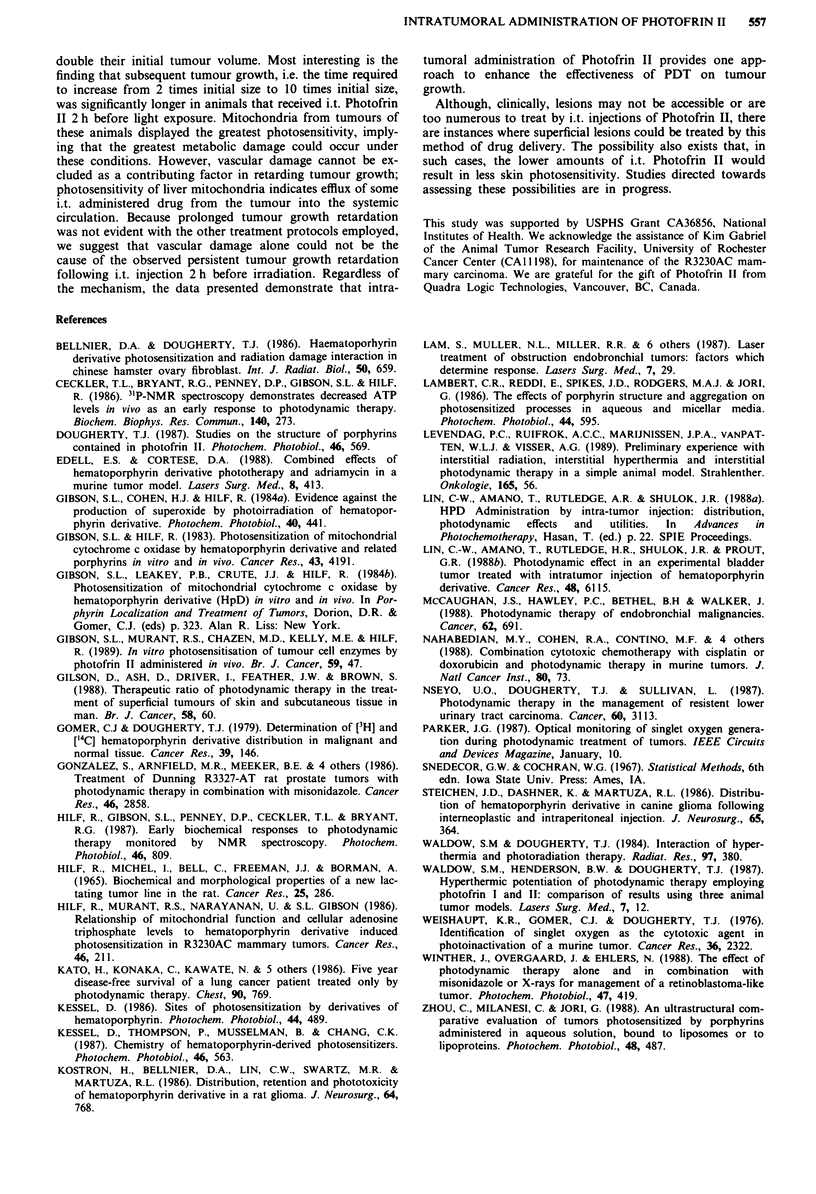# Increased efficacy of photodynamic therapy of R3230AC mammary adenocarcinoma by intratumoral injection of Photofrin II.

**DOI:** 10.1038/bjc.1990.124

**Published:** 1990-04

**Authors:** S. L. Gibson, K. R. van der Meid, R. S. Murant, R. Hilf

**Affiliations:** Department of Biochemistry, University of Rochester School of Medicine and Dentistry, NY 14642.

## Abstract

Photodynamic therapy consists of the systemic administration of a derivative of haematoporphyrin (Photofrin II) followed 24-72 h later by exposure of malignant lesions to photoradiation. We investigated the efficacy of this treatment after direct intratumoral injection of Photofrin II. This direct treatment regimen resulted in higher rates of inhibition of mitochondrial cytochrome c oxidase (5.13% J-1 cm-2 x 10(-1) and succinate dehydrogenase (3.14% J-1 cm-2 x 10(-1] in vitro at 2 h after intratumoral injection compared to rates of inhibition obtained after intraperitoneal drug administration: 0.51 and 0.42% J-1 cm-2 x 10(-1), respectively. A significant delay in tumour growth in vivo was observed in animals that received intratumoral injections 2 h before photoradiation compared to animals injected intraperitoneally at either 2 or 24 h before photoradiation. The treatment protocols were compared with control groups, consisting of Photofrin II administration intratumorally or intraperitoneally without photoradiation, or photoradiation in the absence of Photofrin II. These data indicate that the intratumoral injection regimen with Photofrin II enhanced the efficacy of photodynamic therapy. The greater delay in tumour growth observed after intratumoral administration of Photofrin II suggests a mechanism favouring direct cell damage.


Br. J. Cancer (1990), 61, 553-557 © Macmillan Press Ltd., 1990

### Increased efficacy of photodynamic therapy of R3230AC mammary adenocarcinoma by intratumoral injection of Photofrin II

S.L. Gibson, K.R. Van Der Meid, R.S. Murant & R. Hilf

*Department of Biochemistry and the UR Cancer Center, Box 607, University of Rochester School of Medicine and Dentistry, 601 Elmwood Avenune, Rochester, NY 14642, USA.*

**Summary** Photodynamic therapy consists of the systemic administration of a derivative of haematoporphyrin (Photofrin II) followed 24–72 h later by exposure of malignant lesions to photoradiation. We investigated the efficacy of this treatment after direct intratumoral injection of Photofrin II. This direct treatment regimen resulted in higher rates of inhibition of mitochondrial cytochrome c oxidase (5.13% $J^{-1}\,cm^{-2} \times 10^{-1}$) and succinate dehydrogenase (3.14% $J^{-1}\,cm^{-2} \times 10^{-1}$) *in vitro* at 2 h after intratumoral injection compared to rates of inhibition obtained after intraperitoneal drug administration: 0.51 and 0.42% $J^{-1}\,cm^{-2} \times 10^{-1}$, respectively. A significant delay in tumour growth *in vivo* was observed in animals that received intratumoral injections 2 h before photoradiation compared to animals injected intraperitoneally at either 2 or 24 h before photoradiation. The treatment protocols were compared with control groups, consisting of Photofrin II administration intratumorally or intraperitoneally without photoradiation, or photoradiation in the absence of Photofrin II. These data indicate that the intratumoral injection regimen with Photofrin II enhanced the efficacy of photodynamic therapy. The greater delay in tumour growth observed after intratumoral administration of Photofrin II suggests a mechanism favouring direct cell damage.

Photodynamic therapy (PDT), a promising therapeutic modality for the management of various types of malignancies, employs a combination of the systemic administration of a photosensitiser (Photofrin II) with the direct exposure of tumours to visible irradiation, a protocol that results in metabolic inhibition of malignant cells *in vitro* and *in vivo* (Hilf *et al.*, 1986, 1987; Kessel, 1986; Ceckler *et al.*, 1986). The photosensitised damage is attributed to production of the highly reactive oxygen species, singlet oxygen, which is formed upon exposure of the porphyrin components in Photofrin II to visible light (Weishaupt *et al.*, 1976; Gibson *et al.*, 1984*a*; Parker, 1987). Two features of PDT are noteworthy. The hydrophobic components (Dougherty, 1987; Kessel *et al.*, 1987) of Photofrin II (presumably di-haematoporphyrin ethers and/or esters) are retained for longer periods in tumour tissue than in most normal tissues (Gomer & Dougherty, 1979; Kostron *et al.*, 1986; Steichen *et al.*, 1986; Lin *et al.*, 1988*a*), resulting in a favourable tumour to normal tissue ratio of photosensitiser. By creating a therapeutic window at selected times after drug administration, irradiation would produce minimal deleterious effects in the surrounding normal tissues due to their reduced porphyrin content. The second feature of PDT is the ability to deliver focused visible light energy via laser fibre optics, thus providing a precise tumour treatment. The typical clinical PDT protocol consists of the systemic administration of 2–5 $mg\,kg^{-1}$ Photofrin II followed 24–72 h later by exposure of malignant lesions to 50–400 $J\,cm^{-2}$ visible photoradiation, usually 630 nm laser emission. Utilising these treatment conditions for various types of malignancies, encouraging clinical results have been reported (Kato *et al.*, 1986; Lam *et al.*, 1987; Gilson *et al.*, 1988; Nseyo *et al.*, 1987; McCaughan *et al.*, 1988). Any improvement in the efficacy of PDT should increase its acceptance as a useful cancer treatment.

Some additional information has been reported recently to improve PDT by modifying the standard treatment protocol. Lin *et al.* (1988*b*) compared effects of PDT after injecting Photofrin II directly into the tumour versus intraperitoneal injection. Although they found no significant difference in the response of a murine bladder tumour, assessed by measuring cell survival *in vitro*, they suggested that the effects observed may occur by different mechanisms, i.e. induction of vascular damage after i.p. administration versus direct cytotoxicity after intratumoral injection (Lin *et al.*, 1988*b*). However, Kostron *et al.* (1986) found an increased efficacy for PDT after intratumoral injection in a rodent glioma. We conducted studies to modify the standard PDT protocol in seeking to increase its efficacy for treatment of a transplantable rodent mammary adenocarcinoma. Data presented here demonstrate that intratumoral administration of Photofrin II increases the efficacy of PDT in this mammary tumour model.

Correspondence: R. Hilf.
Received 11 August 1989; and in revised form 9 November 1989.

### Materials and methods

### *Materials*

All chemicals were purchased from Sigma Chemical Co. (St Louis, MO, USA) unless otherwise noted. Photofrin II, generously provided by Quadra Logic Technologies Inc. (Vancouver, British Columbia, Canada), was received frozen, thawed at room temperature in the dark, divided into 1 ml aliquots, and stored at −70°C until used.

### *Animals and tumours*

The R3230AC mammary adenocarcinoma was maintained by transplantation into the axillary region of 80–100 g female Fischer rats, using the sterile trochar method described earlier (Hilf *et al.*, 1965).

### *In vivo–in vitro protocol*

Photofrin II was administered to tumour-bearing rats, either systemically by intraperitoneal (i.p.) injection or by intratumoral (i.t) inoculation. Each injection was followed by an equilibration period of either 2 or 24 h, during which time tumour-bearing animals were housed in the dark. Intratumoral injections employed in the *in vivo–in vitro* protocol were, depending on the required injection volume, performed with either a 1 ml disposable syringe (> 50 μl) or a Hamilton syringe (< 50 μl), each fitted with a 27 gauge 5/8 inch needle. The needle was inserted laterally at the tumour midline and positioned approximately at the centre of the tumour, where the Photofrin II was injected. Initial tumour volumes before administration of Photofrin II i.t. or i.p. ranged from 0.79 to 2.56 $cm^3$ allowing for a sufficient amount of tissue for preparation of mitochondria. Animals bearing tumours were selected randomly for either i.t. or i.p. administration of Photofrin II, each treatment group consisting of animals with tumours having volumes spanning the above stated range. Photofrin II concentrations administered i.t. were adjusted to attain equivalent body weight (b.w.) doses of 0.25, 0.5, 2.5 or 5.0 mg $kg^{-1}$ for the dose studies and 5.0 mg $kg^{-1}$ for the i.t. versus i.p. comparative studies. The upper limit of tumour volume, 2.56 $cm^3$, used in these studies represents tumours that measured less than 1.5 cm maximum diameter. Based on previous microscopic and magnetic resonance imaging studies of this mammary tumour model, the extent of necrosis in this range is estimated to be less than 10% of tumour volume and is usually focal in nature; it was not thought to alter significantly either the distribution or clearance of the injected Photofrin II. The animals were killed at selected times, tumours and livers were surgically excised in dimmed room light, and suspensions of mitochondria were prepared from whole tissues and stored in 1 ml aliquots at −70°C until assayed (Gibson & Hilf, 1983).

*Photoradiation of mitochondrial suspensions in vitro*

One ml aliquots of tumour or liver mitochondrial suspensions were removed from storage, thawed at room temperature and adjusted to the desired initial enzyme activity by dilution with preparation buffer (see below) before photoradiation of the suspensions *in vitro*. One ml aliquots of these suspensions were exposed to photoradiation emitted from a filtered (570–700 nm) focused quartz halogen light source. The samples placed in 3 ml quartz cuvettes were positioned in the 1 cm diameter focussed beam and irradiated with a power density of 150 mW $cm^{-2}$, measured by a power radiometer (Model Rk 5200, Laser Precision, Utica, NY, USA) connected to an Rk 545 radiometer probe. The suspensions were stirred magnetically and, at selected times, samples (10–40 µl) were removed for analysis of enzyme activity. Temperature of the suspensions, which was monitored during the irradiation period (1 h, 540 J $cm^{-2}$ total fluence), did not rise above ambient (25°C).

*Enzyme activity analysis*

The activities of cytochrome c oxidase and succinate dehydrogenase were analysed at various intervals during the *in vitro* exposure of the mitochondrial suspensions to photoradiation. Before photoradiation, liver or tumour suspensions were adjusted to selected initial enzyme activities by dilution with the preparation buffer (0.33 M sucrose, 1 mM dithiothreitol, 1 mM EGTA, 0.03% bovine serum albumin and 100 mM KCl); these activities were 0.4–0.6 µmol cytochrome c oxidised per min per mg protein for cytochrome c oxidase and 4.6–8.3 × $10^{-2}$ mmol p-iodonitrotetrazolium violet (INT) oxidised per min per mg protein for succinate dehydrogenase.

*Laser photoradiation of tumours in vivo*

Tumours, borne on host animals administered Photofrin II, were photoradiated after reaching a size of 0.4–0.9 $cm^2$ surface area (calculated from two opposing diagonal caliper measurements), which corresponded to a volume range of 0.18–0.54 $cm^3$ (see below) and a final drug dose of 0.81–1.55 mg $kg^{-1}$ body weight (b.w.). Animals were apportioned to each treatment group to provide similar tumour size ranges. The tumour volumes employed in this aspect, involving tumour growth behaviour *in vivo*, were smaller than those used above in the *in vivo–in vitro* protocol. Smaller tumours could be used since it was not necessary to obtain large amounts of tissue for subsequent preparation of subcellular organelles. Further, as tumour size increases, PDT is less effective, presumably due to insufficient light penetration resulting in cytotoxicity only in the outermost regions of the lesion. The tumours were exposed to a 1 cm diameter beam emitted from a fibre optic cable fitted with a cylindrical lens and coupled to an argon pumped tunable dye laser (Coherent, Palo Alto, CA, USA). Power density incident on the tumours was adjusted to 200 mW $cm^{-2}$ as measured using a power radiometer (RK5200, Laser Precision, Utica, NY, USA).

*Tumour volume determinations and examination of treatment efficacy*

Tumour volume was calculated according to $V = r^2\pi H$, where the width, $r$, and the length, $H$, were obtained with calipers. The actual tumour volume was assessed by measuring the water displacement for the whole tumour, and comparing such values with volumes calculated by caliper measurements on the same tumour before its removal from the host. Estimation of volume by use of the equation yielded an average over-estimate of 25% for a cohort of six representative tumours whose volume was obtained by displacement measurements. Nevertheless, growth of each tumour was followed by caliper measurement and the increase in calculated tumour volume is presented as the number of days required for each tumour to reach 2, 5 or 10 times its initial volume. Analyses of the data using designated increments in tumour volume provide a more consistent basis for comparison among groups, particularly when initial tumour volumes, i.e. start of treatment, could vary (usually ±20%) and the initial treatment may have begun at different days after tumour implantation.

*Statistical analysis*

Tukey's multiple comparison procedure (Snedecor & Cochran, 1967) was used to assess significant differences in tumour volume; changes from initial to 2 times initial volume, and from 2 times initial to 10 times initial volume were compared. A value of $P < 0.05$ was considered to be significant.

### Results

*Effects of Photofrin II induced photosensitisation on mitochondrial enzyme activities in vitro following intratumoral drug administration*

Photofrin II, administered i.p., results in a dose dependent inhibition of tumour mitochondrial cytochrome c oxidase and succinate dehydrogenase (SDH) during *in vitro* exposure of mitochondrial suspensions to visible irradiation (Gibson *et al*., 1989). Here we examined whether such a dose relationship existed after Photofrin II was administered intratumorally (i.t.). Two hours before killing, Photofrin II was administered i.t. at doses equivalent to 0.25, 0.5, 2.5 or 5.0 mg $kg^{-1}$, and mitochondria prepared from tumour and liver were exposed to photoradiation (see Materials and methods). The data for cytochrome c oxidase (Figure 1) are presented to demonstrate that both a drug-dose and light-dose relationship existed for the inhibition of this enzyme and for SDH (not shown), in mitochondria prepared from tumours that were injected i.t. with Photofrin II 2 h before killing. Liver mitochondria prepared from the same animals also displayed dose-related inhibitions of both of these enzymes, but at this 2 h time point the extent of inhibition of liver enzymes was considerably less than that observed in tumours (data not shown). The rates of inhibition, calculated as per cent enzyme inhibition per joule per $cm^2$, which were derived from the linear initial portion of the inhibition curves as in Figure 1, are compiled in Table I. The increases in the enzyme inhibition rates were drug-dose dependent, displaying linearity in tumours for i.t. doses up to 2.5 mg $kg^{-1}$ b.w. and for liver up to 5.0 mg $kg^{-1}$ b.w., results suggesting that a maximum tumour porphyrin level was reached by direct injection. A comparison between tumour and liver mitochondrial preparations, at 2 h after i.t. injection, demonstrated that liver was 5–10-fold less susceptible to photosensitisation for each dose of Photofrin II administered. We interpret these results to indicate that higher concentrations of porphyrin were present in tumour tissue at this time, rather than inherent differences in enzyme sensitivity in these two tissues.

**Figure 1** Effects of intratumoral injection of Photofrin II on inhibition of mitochondrial enzymes *in vitro*. Tumour mitochondria were prepared 2 h after i.t. administration of Photofrin II at 0.25 (□,), 0.5 (▲) , 2.5 (○) or 5.0 (■) mg kg$^{-1}$ b.w. and photoradiated *in vitro* with broad band light (570–700 nm) at a power dose of 150 mW cm$^{-2}$. The data are expressed as per cent of initial enzyme activity (zero time before photoradiation) for cytochrome c oxidase in the tumour. Each data point represents the mean of four separate experiments performed in duplicate; error bars are the s.e.m.

Liver and tumour mitochondria from untreated animals were prepared, incubated with Photofrin II *in vitro*, resuspended in buffer after removal of Photofrin II solution and photoradiated. No difference was observed in the photo-induced rate of inhibition of either cytochrome c oxidase or SDH present in either preparation, demonstrating that there were no inherent differences in enzyme sensitivity attributable to tissue source.

### *Comparison of intraperitoneal versus intratumoral administration of Photofrin II on the activities of mitochondrial enzymes in vitro*

Photofrin II (5 mg kg$^{-1}$ b.w.) was administered either i.p. or i.t. at 2 or 24 h before killing of tumour-bearing animals and preparation of mitochondria. The data obtained for the photoradiation-induced inhibition of cytochrome c oxidase and SDH in tumour and liver preparations *in vitro*, presented as the calculated rates of enzyme inhibition, are compiled in Table II. The data clearly demonstrate that photosensitised inhibition of tumour mitochondrial enzymes *in vitro* 2 h after administration of Photofrin II i.t. was much greater than that observed after i.p. administration (5.45 *v.* 0.51% and 3.98 *v.* 0.45% inhibition J$^{-1}$ cm$^{-2}$ × 10$^{-1}$ for cytochrome c oxidase and succinate dehydrogenase, respectively). At 2 h, the liver mitochondrial enzymes also demonstrated a greater enzyme inhibition rate for i.t. *v.* i.p. drug admininstration. In tumour preparations obtained at 24 h post-injection, i.t. administration of Photofrin II continued to be more effective in causing photosensitised inhibition of both enzymes compared to i.p. injection. However, for the liver preparations, obtained 24 h after drug administration, a difference in response of cytochrome c oxidase and SDH relative to route of administration of photosensitiser was no longer apparent.

**Table I** Rates of enzyme inhibition *in vitro* following i.t. administration of Photofrin II *in vivo*

| *Photofrin dose (mg kg$^{-1}$)* | *Cytochrome c oxidase* Tumour | Liver | *Succinate dehydrogenase* Tumour | Liver |
|---|---|---|---|---|
| 0.25 | 1.18±.06 | 0.30±.014 | 1.00±.06 | 0.39±.035 |
| 0.50 | 1.45±.08 | 0.36±.037 | 1.37±.13 | 0.42±.024 |
| 2.50 | 4.24±.36 | 0.57±.05 | 2.70±.36 | 0.66±.054 |
| 5.00 | 5.13±.37 | 0.94±.07 | 3.14±.22 | 0.74±.091 |

Photofrin II was administered by direct tumour injection at various doses: 0.25, 0.5, 2.5 or 5.0 mg kg$^{-1}$ b.w. Tumour and liver mitochondria were prepared 2 h after Photofrin II administration and exposed to broad band illumination (570–700 nm) at a power dose of 150 mW cm$^{-2}$. Rates of enzyme inhibition were calculated from the linear portion of the inhibition curves in Figure 1. Rates are expressed as percent enzyme inhibition J$^{-1}$ cm$^{-2}$ and are presented as means ± s.e.m. Initial activities (0 light) were adjusted by dilution of mitochondria and were: cytochrome c oxidase, 0.4–0.6 μmol cytochrome c oxidised per min per mg protein; succinate dehydrogenase, 4.6–8.3 × 10$^{-2}$ μmol p-iodonitrotetrazolium violet oxidised per min per mg protein.

### *Comparison of effects of intratumoral versus intraperitoneal Photofrin II administration on tumour growth*

Tumour growth was assessed in both treated and untreated animals by determination of tumour volume at regular intervals after tumours became palpable. Analyses of these data, presented as time in days necessary to attain 2, 5 or 10 times initial volume, are displayed in Figure 2. Intratumoral Photofrin II was administered at a dose of 0.5 mg cm$^{-3}$, which represented a range of 0.81–1.55 mg kg$^{-1}$ b.w. Statistical analysis of the data depicted in Figure 2, using Tukey's multiple comparison procedure, indicates that Photofrin II at 0.5 mg cm$^{-3}$ tumour i.t. or at 10 mg kg$^{-1}$ i.p. caused a statistically significant delay of tumour growth when compared to tumour growth in animals injected i.t. with Photofrin II or 10 mg kg $^{-1}$ i.p. but not irradiated (dark controls). This delay in tumour growth in those animals receiving i.t. Photofrin II

**Table II** Comparison of rates of mitochondrial enzyme inhibition following either i.p. or i.t. administration of Photofrin II

| *Enzyme* | *Tissue* | *2 h* i.t. | i.p. | *24 h* i.t. | i.p. |
|---|---|---|---|---|---|
| Cytochrome c oxidase | Tumour | 5.13±0.37 | 0.51±0.034 | 1.11±0.07 | 0.66±0.09 |
| | Liver | 0.94±0.07 | 0.21±0.013 | 0.94±0.11 | 0.72±0.06 |
| Succinate dehydrogenase | Tumour | 3.14±0.22 | 0.42±0.05 | 2.12±0.09 | 1.09±0.04 |
| | Liver | 0.74±0.09 | 0.29±0.016 | 0.67±0.05 | 0.85±0.084 |

Photofrin II was administered either i.p. or i.t. at 5 mg kg$^{-1}$ b.w. at 2 or 24 h before preparation and exposure of tumour or liver mitochondria to irradiation. Photoradiation was performed as described in the Methods. Rates of enzyme inhibition were derived from the linear portion of the inhibition curves displayed in Figure 2. Rates are expressed as per cent enzyme inhibition J$^{-1}$ cm$^{-2}$ × 10$^{-1}$ and are presented as means ± s.e.m. Initial activities (0 light) were the same as listed in Table I.

**Figure 2** The effects of i.t. *v.* i.p. Photofrin II administration on R3230AC tumour growth. Tumours were injected directly with Photofrin II and not exposed to photoradiation (■), or injected i.t. 2 h (▲) or 24 h (●) before light exposure *in vivo*. Comparison was made to tumours borne on animals injected i.p. with 10 mg kg$^{-1}$ i.p. 2 h before photoradiation (△). *In vivo* photoradiation conditions are described in the Methods. Data are expressed as time, in days, required for tumours to attain 2, 5 or 10 times their initial volume, based on measurements obtained before any treatment. Each data point represents the mean tumour volume obtained from six or more tumours; error bars are the s.e.m.

(irradiation 2 h after injection) continued during the period that tumour volume increased from 2 to 10 times initial size ($P = 0.05$ *v.* all other groups). However, the other PDT regimens (Photofrin II administered at 0.5 mg cm$^{-3}$ tumour i.t. 24 h before irradiation or at 10 mg kg$^{-1}$ i.p. 2 h before irradiation) did not significantly alter tumour growth during the latter tumour growth period. Tumours borne on animals that had not received Photofrin II (light control) were irradiated with a total fluence of 360 J cm$^{-2}$ (200 mW cm$^{-2}$ 30 min$^{-1}$) and demonstrated no alteration in tumour growth when compared to untreated controls. Intratumoral temperature measurements were obtained under the above conditions, using a Model 41TD telethermometer (Yellow Springs Instruments, Yellow Springs, OH, USA) connected to a needle probe; the intratumoral temperature did not rise above 39°C. The data represented graphically in Figure 2 demonstrate that the 2 h interval between drug administration i.t. and photoradiation was signficantly more effective in producing delay of tumour growth.

### Discussion

Although combinations of PDT with other treatment modalities, such as *X*-irradiation (Bellnier & Dougherty, 1986; Winther *et al.*, 1988; Levendag *et al.*, 1989), chemotherapy using adriamycin (Edell & Cortese, 1988), cisplatin or doxorubicin (Nahabedian *et al.*, 1988), hyperthermia by combining PDT with microwave irradiation (Waldow & Dougherty, 1984; Waldow *et al.*, 1987; Levendag *et al.*, 1989) and the use of hypoxic cell sensitisers, e.g. misonidazole (Gonzalez *et al.*, 1986; Winther *et al.*, 1988), have been reported, the results have been inconsistent. Less attention has been given to exploring modification of the commonly used PDT protocol so as to improve its efficacy. A number of variables can be investigated including: (i) increasing the concentrations of photosensitiser in neoplastic tissue to give a higher tumour/normal tissue ratio; (ii) light delivery, which optimally should provide sufficient and uniform photon flux throughout the neoplastic tissue; and (iii) adequate oxygen concentrations for production of singlet oxygen throughout the irradiation schedule. Our laboratory and others have begun to address the first of these components, delivery of photosensitiser. Zhou *et al.* (1988) administered haematoporphyrin that was incorporated into liposomes or was bound to lipoproteins of various density to improve the uptake of photosensitiser by tumour tissue after systemic admininstration. With both modified delivery vehicles, tumour response to PDT was more rapid and cell-directed than seen after conventional injection. In several earlier reports (Kostron *et al.*, 1986; Lin *et al.*, 1988*a*, *b*), a direct injection of HpD into tumours was used so as to increase the efficacy of PDT as well as investigate the mechanisms of cell cytotoxicity and tumour regression. Although a transplantable rodent glioma demonstrated a greater responsiveness when HpD was administered i.t. (Kostron *et al.*, 1986), response of the mouse MBT-2 bladder tumour was not enhanced, even though 5–10 times more porphyrin was present in tumours after i.t. injection (Lin *et al.*, 1988 *a*, *b*). Perhaps tumour type and/or host species could account for such apparent differences in response.

In this report, we compared the effects of two routes of drug administration on photosensitisation of mitochondria and on tumour growth after PDT. For study of mitochondrial effects, the *in vivo–in vitro* protocol was employed, an approach that takes into account any host metabolism and intracellular localisation of the photosensitiser occurring *in vivo* (Gibson & Hilf, 1983; Gibson *et al.*, 1984*b*, 1989). From such data, we can derive an estimate of the pharmacokinetics of the administered photosensitiser. Previously, after i.p. administration of either HpD or Photofrin II, a time course of photosensitivity of these organelles *in vitro* indicated that inhibition of mitochondrial function would be greatest when tumours were exposed to light 24–72 h after drug administration (Gibson *et al.*, 1989). Intratumoral administration of Photofrin II, however, showed a different time-course. At 2 h after i.t. administration, we observed a 10-fold greater rate of photosensitiser-induced inhibition of tumour mitochondrial enzymes than that observed in comparable mitochondrial preparations after animals had received equivalent doses of Photofrin II (5 mg kg$^{-1}$) intraperitoneally. By 24 h after Photofrin II administration, such differences in photosensitivity of enzymes narrow considerably, with only 2-fold greater sensitivity for i.t. than for i.p. routes. These results imply that there were higher concentrations of Photofrin II in tumours shortly after i.t. administration, whereas after i.p. administration, the level of porphyrin at 2 or 24 h, based on mitochondrial enzyme inhibition, was largely unchanged.

A different pattern of photosensitisation of liver mitochondria was observed. Surprisingly, liver mitochondria displayed greater photosensitivity at 2 h after i.t. administration than after i.p. injection, as evidenced by the light-induced inhibition of cytochrome c oxidase and SDH activities. This finding suggests that after direct injection into the tumour, more effective levels of drug reached the liver via the systemic circulation than after i.p. injection. One possbile explanation for this observation might be attributed to the presence of multimeric aggregates versus dimeric and monomeric haematoporphyrin species reaching the liver from the peritoneal injection site. While all of these forms after tissue extraction contribute to the amount of drug measured chemically, not all forms give equal singlet oxygen yields upon photoirradiation (Lambert *et al.*, 1986). In contrast, at 24 h, liver mitochondrial enzymes were more photosensitive than at 2 h after i.p. injection, suggesting a greater accumulation and/or a slower efflux of active forms of the photosensitiser. A more extensive study of distribution of photosensitiser in other normal tissues after i.t. injection is warranted.

The results obtained from examination of mitochondrial enzyme inhibition, using the *in vivo–in vitro* protocol, were correlatable to effects of PDT on delay of tumour growth. No significant effects on tumour growth occurred in animals that received Photofrin II (i.p. at 10 mg kg$^{-1}$ or i.t. at 1 mg kg$^{-1}$ b.w.) but were not photoradiated (dark controls), nor in animals that received no drug but light (light controls). In contrast, tumours in animals given Photofrin II i.t. at 1 mg kg$^{-1}$ b.w. and irradiated either at 2 or 24 h later displayed a significant delay in the length of time required to double their initial tumour volume. Most interesting is the finding that subsequent tumour growth, i.e. the time required to increase from 2 times initial size to 10 times initial size, was significantly longer in animals that received i.t. Photofrin II 2 h before light exposure. Mitochondria from tumours of these animals displayed the greatest photosensitivity, implying that the greatest metabolic damage could occur under these conditions. However, vascular damage cannot be excluded as a contributing factor in retarding tumour growth; photosensitivity of liver mitochondria indicates efflux of some i.t. administered drug from the tumour into the systemic circulation. Because prolonged tumour growth retardation was not evident with the other treatment protocols employed, we suggest that vascular damage alone could not be the cause of the observed persistent tumour growth retardation following i.t. injection 2 h before irradiation. Regardless of the mechanism, the data presented demonstrate that intratumoral administration of Photofrin II provides one approach to enhance the effectiveness of PDT on tumour growth.

Although, clinically, lesions may not be accessible or are too numerous to treat by i.t. injections of Photofrin II, there are instances where superficial lesions could be treated by this method of drug delivery. The possibility also exists that, in such cases, the lower amounts of i.t. Photofrin II would result in less skin photosensitivity. Studies directed towards assessing these possibilities are in progress.

This study was supported by USPHS Grant CA36856, National Institutes of Health. We acknowledge the assistance of Kim Gabriel of the Animal Tumor Research Facility, University of Rochester Cancer Center (CA11198), for maintenance of the R3230AC mammary carcinoma. We are grateful for the gift of Photofrin II from Quadra Logic Technologies, Vancouver, BC, Canada.

### References

BELLNIER, D.A. & DOUGHERTY, T.J. (1986). Haematoporhyrin derivative photosensitization and radiation damage interaction in chinese hamster ovary fibroblast. *Int. J. Radiat. Biol.*, **50**, 659.

CECKLER, T.L., BRYANT, R.G., PENNEY, D.P., GIBSON, S.L. & HILF, R. (1986). $^{31}$P-NMR spectroscopy demonstrates decreased ATP levels *in vivo* as an early response to photodynamic therapy. *Biochem. Biophys. Res. Commun.*, **140**, 273.

DOUGHERTY, T.J. (1987). Studies on the structure of porphyrins contained in photofrin II. *Photochem. Photobiol.*, **46**, 569.

EDELL, E.S. & CORTESE, D.A. (1988). Combined effects of hematoporphyrin derivative phototherapy and adriamycin in a murine tumor model. *Lasers Surg. Med.*, **8**, 413.

GIBSON, S.L., COHEN, H.J. & HILF, R. (1984*a*). Evidence against the production of superoxide by photoirradiation of hematoporphyrin derivative. *Photochem. Photobiol.*, **40**, 441.

GIBSON, S.L. & HILF, R. (1983). Photosensitization of mitochondrial cytochrome c oxidase by hematoporphyrin derivative and related porphyrins *in vitro* and *in vivo*. *Cancer Res.*, **43**, 4191.

GIBSON, S.L., LEAKEY, P.B., CRUTE, J.J. & HILF, R. (1984*b*). Photosensitization of mitochondrial cytochrome c oxidase by hematoporphyrin derivative (HpD) *in vitro* and *in vivo*. In *Porphyrin Localization and Treatment of Tumors*, Dorion, D.R. & Gomer, C.J. (eds) p. 323. Alan R. Liss: New York.

GIBSON, S.L., MURANT, R.S., CHAZEN, M.D., KELLY, M.E. & HILF, R. (1989). *In vitro* photosensitisation of tumour cell enzymes by photofrin II administered *in vivo*. *Br. J. Cancer*, **59**, 47.

GILSON, D., ASH, D., DRIVER, I., FEATHER, J.W. & BROWN, S. (1988). Therapeutic ratio of photodynamic therapy in the treatment of superficial tumours of skin and subcutaneous tissue in man. *Br. J. Cancer*, **58**, 60.

GOMER, C.J & DOUGHERTY, T.J. (1979). Determination of [$^{3}$H] and [$^{14}$C] hematoporphyrin derivative distribution in malignant and normal tissue. *Cancer Res.*, **39**, 146.

GONZALEZ, S., ARNFIELD, M.R., MEEKER, B.E. & 4 others (1986). Treatment of Dunning R3327-AT rat prostate tumors with photodynamic therapy in combination with misonidazole. *Cancer Res.*, **46**, 2858.

HILF, R., GIBSON, S.L., PENNEY, D.P., CECKLER, T.L. & BRYANT, R.G. (1987). Early biochemical responses to photodynamic therapy monitored by NMR spectroscopy. *Photochem. Photobiol.*, **46**, 809.

HILF, R., MICHEL, I., BELL, C., FREEMAN, J.J. & BORMAN, A. (1965). Biochemical and morphological properties of a new lactating tumor line in the rat. *Cancer Res.*, **25**, 286.

HILF, R., MURANT, R.S., NARAYANAN, U. & S.L. GIBSON (1986). Relationship of mitochondrial function and cellular adenosine triphosphate levels to hematoporphyrin derivative induced photosensitization in R3230AC mammary tumors. *Cancer Res.*, **46**, 211.

KATO, H., KONAKA, C., KAWATE, N. & 5 others (1986). Five year disease-free survival of a lung cancer patient treated only by photodynamic therapy. *Chest*, **90**, 769.

KESSEL, D. (1986). Sites of photosensitization by derivatives of hematoporphyrin. *Photochem. Photobiol.*, **44**, 489.

KESSEL, D., THOMPSON, P., MUSSELMAN, B. & CHANG, C.K. (1987). Chemistry of hematoporphyrin-derived photosensitizers. *Photochem. Photobiol.*, **46**, 563.

KOSTRON, H., BELLNIER, D.A., LIN, C.W., SWARTZ, M.R. & MARTUZA, R.L. (1986). Distribution, retention and phototoxicity of hematoporphyrin derivative in a rat glioma. *J. Neurosurg.*, **64**, 768.

LAM, S., MULLER, N.L., MILLER, R.R. & 6 others (1987). Laser treatment of obstruction endobronchial tumors: factors which determine response. *Lasers Surg. Med.*, **7**, 29.

LAMBERT, C.R., REDDI, E., SPIKES, J.D., RODGERS, M.A.J. & JORI, G. (1986). The effects of porphyrin structure and aggregation on photosensitized processes in aqueous and micellar media. *Photochem. Photobiol.*, **44**, 595.

LEVENDAG, P.C., RUIFROK, A.C.C., MARIJNISSEN, J.P.A., VANPATTEN, W.L.J. & VISSER, A.G. (1989). Preliminary experience with interstitial radiation, interstitial hyperthermia and interstitial photodynamic therapy in a simple animal model. Strahlenther. *Onkologie*, **165**, 56.

LIN, C-W., AMANO, T., RUTLEDGE, A.R. & SHULOK, J.R. (1988*a*). HPD Administration by intra-tumor injection: distribution, photodynamic effects and utilities. In *Advances in Photochemotherapy*, Hasan, T. (ed.) p. 22. SPIE Proceedings.

LIN, C.-W., AMANO, T., RUTLEDGE, H.R., SHULOK, J.R. & PROUT, G.R. (1988*b*). Photodynamic effect in an experimental bladder tumor treated with intratumor injection of hematoporphyrin derivative. *Cancer Res.*, **48**, 6115.

McCAUGHAN, J.S., HAWLEY, P.C., BETHEL, B.H & WALKER, J. (1988). Photodynamic therapy of endobronchial malignancies. *Cancer*, **62**, 691.

NAHABEDIAN, M.Y., COHEN, R.A., CONTINO, M.F. & 4 others (1988). Combination cytotoxic chemotherapy with cisplatin or doxorubicin and photodynamic therapy in murine tumors. *J. Natl Cancer Inst.*, **80**, 73.

NSEYO, U.O., DOUGHERTY, T.J. & SULLIVAN, L. (1987). Photodynamic therapy in the management of resistent lower urinary tract carcinoma. *Cancer*, **60**, 3113.

PARKER, J.G. (1987). Optical monitoring of singlet oxygen generation during photodynamic treatment of tumors. *IEEE Circuits and Devices Magazine*, January, 10.

SNEDECOR, G.W. & COCHRAN, W.G. (1967). *Statistical Methods*, 6th edn. Iowa State Univ. Press: Ames, IA.

STEICHEN, J.D., DASHNER, K. & MARTUZA, R.L. (1986). Distribution of hematoporphyrin derivative in canine glioma following interneoplastic and intraperitoneal injection. *J. Neurosurg.*, **65**, 364.

WALDOW, S.M & DOUGHERTY, T.J. (1984). Interaction of hyperthermia and photoradiation therapy. *Radiat. Res.*, **97**, 380.

WALDOW, S.M., HENDERSON, B.W. & DOUGHERTY, T.J. (1987). Hyperthermic potentiation of photodynamic therapy employing photofrin I and II: comparison of results using three animal tumor models. *Lasers Surg. Med.*, **7**, 12.

WEISHAUPT, K.R., GOMER, C.J. & DOUGHERTY, T.J. (1976). Identification of singlet oxygen as the cytotoxic agent in photoinactivation of a murine tumor. *Cancer Res.*, **36**, 2322.

WINTHER, J., OVERGAARD, J. & EHLERS, N. (1988). The effect of photodynamic therapy alone and in combination with misonidazole or X-rays for management of a retinoblastoma-like tumor. *Photochem. Photobiol.*, **47**, 419.

ZHOU, C., MILANESI, C. & JORI, G. (1988). An ultrastructural comparative evaluation of tumors photosensitized by porphyrins administered in aqueous solution, bound to liposomes or to lipoproteins. *Photochem. Photobiol.*, **48**, 487.